# Detecting the Immune System Response of a 500 Year-Old Inca Mummy

**DOI:** 10.1371/journal.pone.0041244

**Published:** 2012-07-25

**Authors:** Angelique Corthals, Antonius Koller, Dwight W. Martin, Robert Rieger, Emily I. Chen, Mario Bernaski, Gabriella Recagno, Liliana M. Dávalos

**Affiliations:** 1 Department of Sciences, John Jay College of Criminal Justice, City University of New York, New York, New York, United States of America; 2 The Proteomics Center at Stony Brook, SUNY Stony Brook Medical Center, Stony Brook, New York, United States of America; 3 Division of Hematology, Department of Medicine, SUNY Stony Brook Medical Center, Stony Brook, New York, United States of America; 4 Department of Pharmacological Sciences, SUNY Stony Brook Medical Center, Stony Brook, New York, United States of America; 5 Museo de Arqueologia de Alta Montaña (MAAM), Salta, Argentina; 6 Department of Ecology and Evolution and Consortium for Inter-Disciplinary Environmental Research, SUNY Stony Brook, Stony Brook, New York, United States of America; 7 Department of Pathology, SUNY Stony Brook Medical Center, Stony Brook, New York, United States of America; University of Calgary & ProvLab Alberta, Canada

## Abstract

Disease detection in historical samples currently relies on DNA extraction and amplification, or immunoassays. These techniques only establish pathogen presence rather than active disease. We report the first use of shotgun proteomics to detect the protein expression profile of buccal swabs and cloth samples from two 500-year-old Andean mummies. The profile of one of the mummies is consistent with immune system response to severe pulmonary bacterial infection at the time of death. Presence of a probably pathogenic *Mycobacterium sp*. in one buccal swab was confirmed by DNA amplification, sequencing, and phylogenetic analyses. Our study provides positive evidence of active pathogenic infection in an ancient sample for the first time. The protocol introduced here is less susceptible to contamination than DNA-based or immunoassay-based studies. In scarce forensic samples, shotgun proteomics narrows the range of pathogens to detect using DNA assays, reducing cost. This analytical technique can be broadly applied for detecting infection in ancient samples to answer questions on the historical ecology of specific pathogens, as well as in medico-legal cases when active pathogenic infection is suspected.

## Introduction

Over the last decade, forensic techniques relying on ancient DNA extraction and PCR amplification have provided critical evidence to resolve longstanding historical questions, such as uncovering pathologies linked to the early death of Tutankhamen [1], or identifying the presence of the pathogen *Yersinia pestis* in bodies excavated from medieval cemeteries [2], [3]. Because extraneous DNA can be easily amplified during PCR, forensic applications rely on strict controls to avoid false positives [4], [5]. When used to infer infection in historical samples, DNA techniques can confirm pathogen presence but cannot positively infer disease because a pathogen could be present without causing infection [6], [7], [8]. Such applications are particularly valuable in an archeological context, in which differentiating between natural and deliberate causes of death can significantly change the interpretation of a historical event [1], [2]. Detection of a pathogen, however, is necessary but not sufficient to determine disease because the pathogen could be present without causing infection [6], [7], [8].

Detecting the immune reaction to the pathogen in the host provides positive evidence of active pathogenic infection [9]. Existing methods, such as antibody-binding immunoassays, are ill suited for archeological applications because they require fresh tissues, use a small number of targeted antibodies, and are prone to both false positives and false negatives [10], [11]. Proteomics approaches can identify and quantify proteins directly, and offer three distinct advantages in archeological and forensic research [12]. First, proteins can potentially outlast DNA by thousands to millions of years [13], [14], pushing back the time frame for detection of responses to infection. Second, protein detection does not rely on amplification, so there is less susceptibility to contamination than in PCR [15]. Third, a broad spectrum of proteins can be characterized from small samples, resulting in a more resolved picture of immune response than from immunoassays [16]. In this paper, we present methods for obtaining proteomic-quality samples from 500-year old Andean mummies, and results documenting immune response in these ancient human samples. Our results show that shotgun proteomic applications complement results from forensic DNA analyses by providing evidence of active infection and pointing to the pathogens triggering observed immune responses.

## Methods

### Archaeological Context

**Figure 1:**
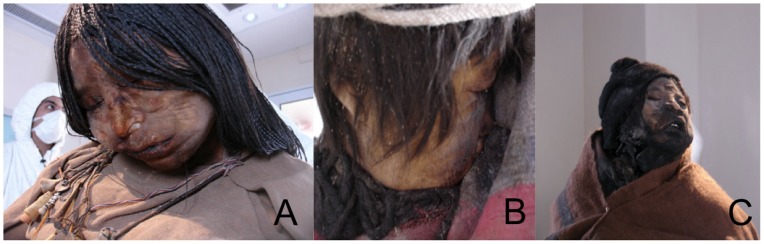
The children of Llullallaico. a) La Doncella (the Maiden); b) El Niño (the Boy); and c) La Niña (the Girl).

In 1999, a team of archaeologists led by Johan Reinhard and Constanza Ceruti, uncovered the site of three burials 25 m from the 6,739-m summit of Llullaillaco, a high elevation volcano in the province of Salta, Argentina. The expedition recovered the preserved bodies of two young children (a 7 year-old boy and a 6-year old girl) and one 15-year old adolescent girl known as “the Maiden”. The three children had been sacrificed to Pachamama, the earth goddess, in the ritual of Capacocha [17], [18], [19]. The outstanding condition of the mummies (fig. 1) was the result of the combination of freezing temperature, mild humidity, anaerobic environment and the presence of natural disinfectants. The bodies were buried about 50 cm underground, and the empty space within the tombs was packed with volcanic ash. The ash inhibited the growth of decomposing bacteria and fungi, and acted as a barrier to moisture, protecting the bodies from external humidity while preserving internal moisture. This atmosphere provided the conditions for the subcutaneous fat of the bodies to transform into soap in a process called adipocere [19], [20]. Finally, a layer of packed snow rendered the tombs airtight shortly after their closing. As a result, the bodies were exceptionally preserved and provided more high-quality physical evidence for their state at the time of death than comparable finds from that period anywhere in the world.

### Sampling

All three Llullaillaco mummies are preserved at Museum of High Mountain Archaeology (MAAM) in Salta (Argentina). They are in airtight, self-contained capsules and maintained at −20°C, in a mix of liquid nitrogen vapor and 2% oxygen. Sampling took place in the cold laboratory adjacent to the mummies’ repository, at −5°C. We sampled a small, blood-soaked piece of cloth from the boy’s cloak, against which his mouth rested. We took four contact mouth swabs from the lips of the Maiden and the boy, since the lips of both presented blood and saliva deposits. The mummy of the young girl (“La Niña”) showed signs of having been struck by lightning (fig. 1) and was not sampled. All samples were placed dry in individual sterile and sealed vials to prevent contamination. They were kept dry at room temperature to avoid any oxidative or hydrolytic lesions to the DNA. The samples were shipped and maintained dry until analyses.

### Proteomic Sample Preparation and Analysis

Three samples were obtained from the mummies: 1) a 3 mm^2^ piece of fabric from the boy, 2) a cotton swab from the lips of the boy, and 3) a cotton swab from the lips of the Maiden. All were processed with the same protocol. The excised tips of the cotton swabs and the fabric were cut off and placed in separate low-protein binding 1.5 ml polypropylene microfuge tubes. All sample tubes received 50 mM NH_4_HCO_3_ sufficient to cover the sample and incubated at 23°C for 10 min followed by 10 min submersion in a bath sonicator at 23°C. The samples were centrifuged for 5 min at 16,000 G and 23°C, and the supernatant transferred to fresh tubes. The moist fabric and cotton were transferred to 500-µl polypropylene tubes perforated with a 22-gauge needle hole in the bottom. The tubes were place into the original 1.5 ml tubes and the combined tubes centrifuged for 1 min at 16,000 G. The passed-through buffer was combined with the removed supernatants. The tubes containing the supernatant and pass-through were centrifuged at 16,000 G for 5 min and the resultant supernatants (∼100 µl) transferred to fresh tubes. The volume of supernatants was reduced to 20 µl using a Speed-Vac, and each tube subsequently received 20 µl of ACN. The samples were reduced by the addition of 1 µl of 0.1 M DTT and incubated 30 min at 23°C. The samples were alkylated by the addition of 1 µl of 0.2 M iodoacetamide and incubated for 30 min at 23°C in the dark. Each tube then received 10 µl of 5X Invitrosol followed by 1 µl of trypsin at 1 mg/ml. The samples were incubated overnight at 37°C. Following incubation, the samples were centrifuged at 16,000 G for 5 min, the supernatants transferred to fresh tubes, and the volumes reduced to 20 µl in a Speed-Vac. Each tube received 5 µl of 0.1% TFA and sufficient volume of 2% (v/v) acetonitrile, 0.2% formic acid to bring the total volume to ∼50 µl. Each sample was divided into 3 ∼ 15-µl aliquots. One aliquot was subjected to immediate mass spectrometry (MS) analysis, while the others were quick-frozen in liquid N_2_ and stored at −80°C.

Fifteen µl of the peptide mixture from each residual sample was analyzed by automated microcapillary liquid chromatography-tandem mass spectrometry on a Thermo LTQ-Orbitrap XL mass spectrometer. Fused-silica capillaries (100 µm i.d.) were pulled using a P-2000 CO_2_ laser puller (Sutter Instruments, Novato, CA) to a 5-µm i.d. tip and packed with 10 cm of 5-µm Magic C18 material (Agilent, Santa Clara, CA) using a pressure bomb. This column was then placed in-line with an Eksigent 2D HPLC with autosampler. The column was equilibrated in buffer A (2% acetonitrile, 0.1% formic acid), and the peptide mixture was loaded onto the column using the autosampler. The HPLC separation at a flow rate of 300 nl/min was provided by a gradient between Buffer A and Buffer B (98% acetonitrile, 0.1% formic acid). The HPLC gradient was held constant at 100% buffer A for 5 min after peptide loading, followed by a 30-min gradient from 5% buffer B to 40% buffer B. Then, the gradient was switched from 40% to 80% buffer B over 5 min and held constant for 3 min. Finally, the gradient was changed from 80% buffer B to 100% buffer A over 1 min, and then held constant at 100% buffer A for 15 more minutes. The application of a 1.8-kV distal voltage electro-sprayed the eluted peptides directly into the mass spectrometer equipped with a custom nanoLC electrospray ionization source. Full mass spectra (MS) were recorded on the peptides over a 400–2000 m/z range at 60,000 resolution (at m/z 400), followed by five tandem mass (MS/MS) events sequentially generated in a data-dependent manner on the first, second, third, fourth and fifth most intense ions selected from the full MS spectrum (at 35% collision energy). Mass-spectrometer scan functions and HPLC solvent gradients were controlled by the Xcalibur data system (ThermoFinnigan, San Jose, CA).

Tandem mass spectra were extracted from raw files with the program RawXtract (fields.scripps.edu). The spectra were searched against a human protein database containing 87,061 protein sequences downloaded as FASTA-formatted sequences from EBI-IPI (database version 3.68) [21] and 54 common contaminant proteins, for a total of 87,115 target database sequences. To calculate confidence levels and false positive rates, a decoy database containing the reverse sequences of 87,115 proteins appended to the target database [22] and the SEQUEST algorithm [23] was used to find the best matching sequences from the combined database. The peptide mass search tolerance was set to 50 ppm. A static modification on cysteines of 57.02146 Da was included. No enzymatic cleavage conditions were imposed on the database search, so the search space included all candidate peptides whose theoretical mass fell within the mass tolerance window, despite their tryptic status. DTASelect [24] was used to filter good peptide matches from the SEQUEST result. Table S1 a full list of the proteins and peptides detected.

### Quantitative Analyses of Proteomic Profiles

A key challenge in analyzing proteomic profiles is identifying adequate controls to establish correspondence with a particular response, or departure from a baseline state. This challenge is magnified for ancient samples, as differential protein degradation could contribute to generating profiles significantly different from current healthy or infected samples. To determine that the expression profile consistent with active infection was not the result of differential protein degradation, we used the expression profile of the boy as a control. We used the spectral counts of the cloth sample from the boy because many more proteins were recovered from this sample than from the boy’s mouth swab (table S2). We did not assume the boy’s sample represented a healthy individual because that mummy showed signs of trauma and bleeding. However, the boy showed no signs of respiratory disease (see below), and therefore contrasted with the Maiden in that respect.

To compare the samples we used nonparametric statistics, as the frequency distributions of spectral counts were highly skewed and there was no basis for computing expected spectral frequencies (e.g., [25], [26]). We divided the proteins recovered into two categories: those involved in inflammatory and immune response, and all others. The spectral counts for the Maiden and the boy were then compared using the Wilcoxon Mann-Whitney test [27], with exact computation of the null distribution of the *Z* statistic and breaking observed rank ties. The test was implemented in the *wilcox*_*test* routine in the coin v.1.0-20 [28] R [29] package. If the proteomic profile of the Maiden corroborated respiratory infection, then the spectral counts of inflammatory and immune response proteins should be significantly elevated relative to the sample from the boy. A similar comparison of other proteins should not be significant.

### DNA Extraction, Amplification, Sequencing and Analyses

All DNA extractions were conducted in a laboratory that undergoes regular decontamination with UV-irradiation and hypochlorite treatment. Each sample extraction was conducted separately to prevent cross-contamination. All extractions were performed in a BSL-II cabinet, which was UV-irradiated for 1 hour prior to each sample extraction. All consumables, including pipettor tips, micro-centrifuge tubes and collection tubes as well as the small equipment such as pipettors were UV-irradiated in a UV crosslinker for 20 minutes at 1200×100 µJ/cm^2^. Gloves were also changed between every step of the extraction to prevent contamination. Mock DNA extractions and control blank PCRs were performed for every DNA assay in the laboratory and screened for contamination.

**Swab sample extraction protocol 1:** two swab tips, one each from the Maiden and the boy were placed in 1.2-ml micro-centrifuge tubes, and DNA was extracted using a modified QIAmp extraction protocol. The swab tips were lysed at 56°C for 60 minutes in 190 µl of QIAmp micro kit ATL buffer (Qiagen Inc., Valencia, CA). We then added 200 µl of AL buffer with 1 µl of Carrier RNA, and incubated the solution at 95°C for 5 minutes in a thermal mixer shaking at 900 rpm. All swab tips were then removed from the micro-centrifuge tubes, and the solution was purified using the QIAmp micro-columns. The samples were eluted using PCR-grade water and stored at 4°C prior to amplification.

**Swab sample extraction protocol 2:** small subsamples (1 mm^3^) of the two swabs from the Maiden and the boy were processed using the ZyGEM forensicGEM Saliva kit (ZyGEM corp. ltd., Solana Beach, CA). The subsamples were washed with DNA-free water, following the forensicGEM saliva kit protocol. The eluates were then transferred to a 0.2 mL PCR clean tube with 10 µl of 10x ZyGEM buffer, 69 µl of DNA-free water and 1 µl of forensicGEM gold buffer. The solution was incubated at 75°C for 15 minutes, then at 95°C for 5 minutes. The samples were then ready for amplification.

A reading of the final DNA concentration for all samples using the Thermo Scientific NanoDrop 1000 spectrophotometer (Thermo Fisher Scientific, ltd., Waltham, MA) was performed to ensure sufficient DNA yield prior amplification.

**Figure 2:**
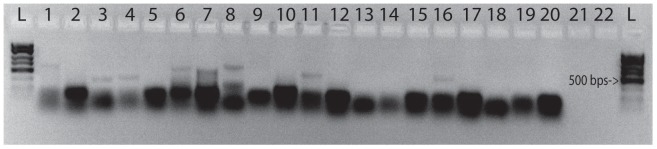
Gel electrophoresis from the Maiden. Gel electrophoresis (2%) showing the amplification of the *Mycobacterium sp. hsp65* gene fragments in the Maiden’s buccal swab samples 6 and 11, both sequenced successfully. The image was inverted to facilitate detection of bands and no other image treatment was performed. Sample 16 did not produce a satisfactory sequence. Samples 3 and 4 were positive for primers described as specific to *Mycobacterium avium*, but did not produce satisfactory sequences. Sample 1 and 7 were positive for primers described as specific to *Mycobacterium tuberculosis*, but did not produce satisfactory sequences. The negative controls are in lanes 17, 18, 19 and 20. Ladders are 100-bp Fisher exACTgene.

**Figure 3:**
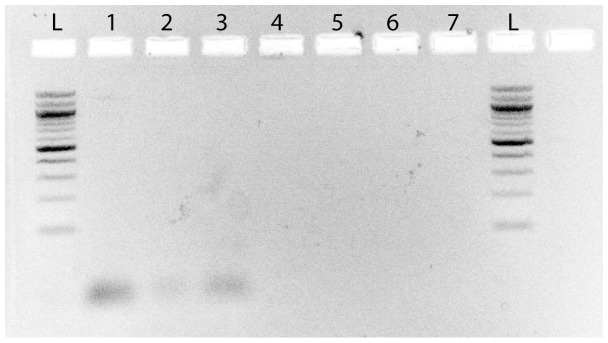
Gel electrophoresis from the boy. Gel electrophoresis (2%) showing the products of PCR amplification from samples from the boy. The results were negative: no amplification bands could be detected. The image was inverted, and contrast was increased to facilitate detection of bands. No other image treatment was performed. Lanes 1 and 2 were amplified with *M. tuberculosis*-specific primers, lanes 3 and 4 with the *Mycobacterium*-specific primers, and lanes 5, 6 and 7 were negative controls.

We used 4 different sets of primers in PCR amplifications from all swab samples, targeting the 16S rRNA, *MTP40* and *hsp65* genes of *Mycobacterium sp., Mycobacterium avium* and *Mycobacterium tuberculosis* (see table 1) [30], [31]. Amplifications were carried out in 25-µl volumes using the PuReTaq Ready-To-Go PCR Beads (GE Healthcare Life Sciences, Pittsburgh, PA). A 5-µl sample of the DNA eluates were added to a solution containing 18 µl of water, 1 µl of each primer and the PuReTaq bead. Three µl of each PCR was stained with ethidium bromide, electrophoresed in 2% agarose for 40 minutes at 20 v/cm, and visualized under UV-light. None of the negative controls amplified (figs. 2–3). All PCR-products were then purified using ExoSap-IT (Affymetrix Inc., Santa Clara, CA). Out of 16 PCR reactions for the Maiden, 8 were positive (see fig. 2). Out of 7 PCR reactions for the boy, all were negative (see fig. 3). All products were sequenced using the ABI prism BigDye Terminator Cycle Sequencing kit (Applied Biosystems, Carlsbad, CA) and analyzed on an ABI 377A automated sequencer. Of the 8 positive PCR reactions, 2 were successfully sequenced (PCR samples 6 and 11; see fig. 2). Sample 6 and 11, both from the Maiden, were amplified and successfully sequenced from two of the ZyGEM-extracted buccal samples. Sample 11 yielded a 275-bp *Mycobacterium sp.* 65-kDa heat-shock protein gene sequence, and sample 6 yielded a 440-bp *Bifidobacterium sp.* 65-kDa heat-shock protein gene sequence.

**Table 1:** Primers used for PCR amplification from DNA extracted from swab samples.

Name	Primer sequence	Prod. Size (bp)	Organism	Region	References
PANMYCF	TGGATCCGACGAAGTCGTAACAAGG	270–400	Genus-wide *Mycobacterium*	16S rRNA	31
PANMYCR	TGGATAGTGGTTGCGAGCAT	270–401	Genus-wide *Mycobacterium*	16S rRNA	31
MACF	CCCTGAGACAACACTCGGTC	144	*Mycobacterium avium*–specific	16S rRNA	31
MACR	GTTCATCGAAATGTGTAATT	144	*Mycobacterium avium*–specific	16S rRNA	31
Tb-A	CTCGTCCAGCGCCGCTTCGG	123	*Mycobacterium tuberculosis*specific	*MTP40*	30
Tb-B	CCTGCGAGCGTAGGCGTCGG	123	*Mycobacterium tuberculosis*specific	*MTP41*	30
Tb 11	ACCAACGATGGTGTGTCCAT	441	Genus-wide *Mycobacterium*	Heat-shock protein 65 (*hsp65*)	30
Tb 12	CTTGTCGAACCGCATACCCT	441	Genus-wide *Mycobacterium*	Heat-shock protein 65 (*hsp65*)	30

**Figure 4:**
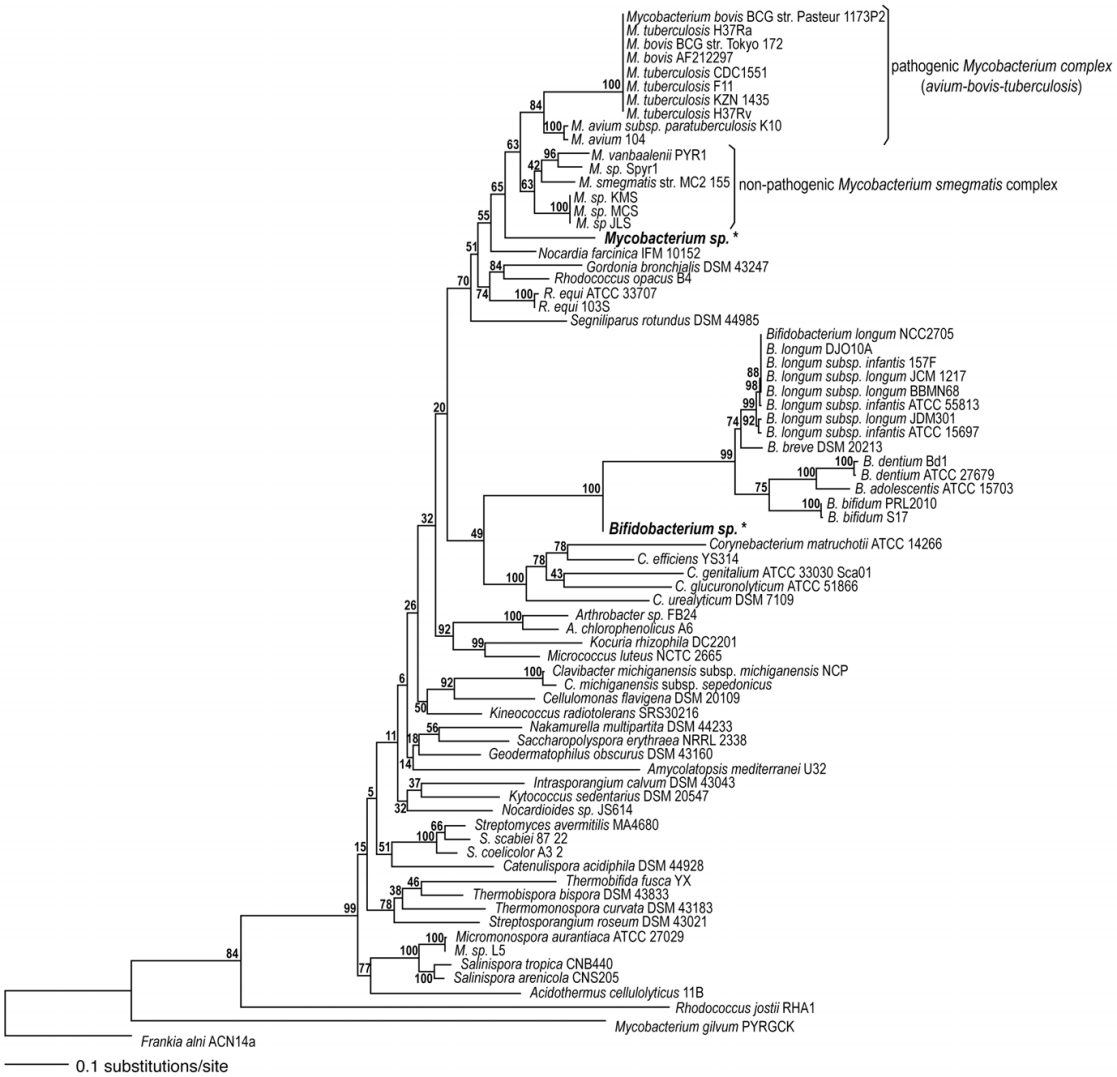
Maximum likelihood phylogeny. Maximum likelihood phylogeny and bootstrap support values based on 1000 pseudoreplicates of the alignment of *hsp65* gene nucleotide sequences. Sequences generated from the Maiden’s swab samples are shown in bold, larger font, and marked with an asterisk.

We identified these sequences using a phylogenetic approach. After initial queries to GenBank matched accessions in the phylum Actinobacteria only, the bidirectional consensus of each of the two fragments was matched against the NCBI reference genomes of Actinobacteria (http://www.ncbi.nlm.nih.gov/sutils/genom_table.cgi) using BLAST [32]. The sequences significantly matched accessions in each case (e-value ≤5e-140 for a 440-bp sequence, and e-value ≤3e-81 for a 276-bp sequence). DNA sequences corresponding to the 65-kDa heat-shock protein genes from the top 50 hits were downloaded and aligned using the *linsi* algorithm with 1000 iterations in mafft v6.710b [33], [34]. The inclusion of both sets of 50 top hits resulted in an alignment of 74 sequences across 1,759 nucleotides, including the two fragments amplified. This alignment was analyzed using the high-performance computing maximum likelihood algorithm on RAxML v7.0.4 [35], [36], and applying a general time reversible model of nucleotide evolution [37] with a discrete approximation to the shape of a continuous gamma distribution for variable rates of change across the alignment [38]. The full alignment was resampled 1000 times to generate bootstrap branch support values [39]. Many accessions were found to be identical, so the unique-sequence subset of 63 taxa was resampled 100 times to generate the phylogeny used in comparisons (fig. 4).

**Figure 5:**
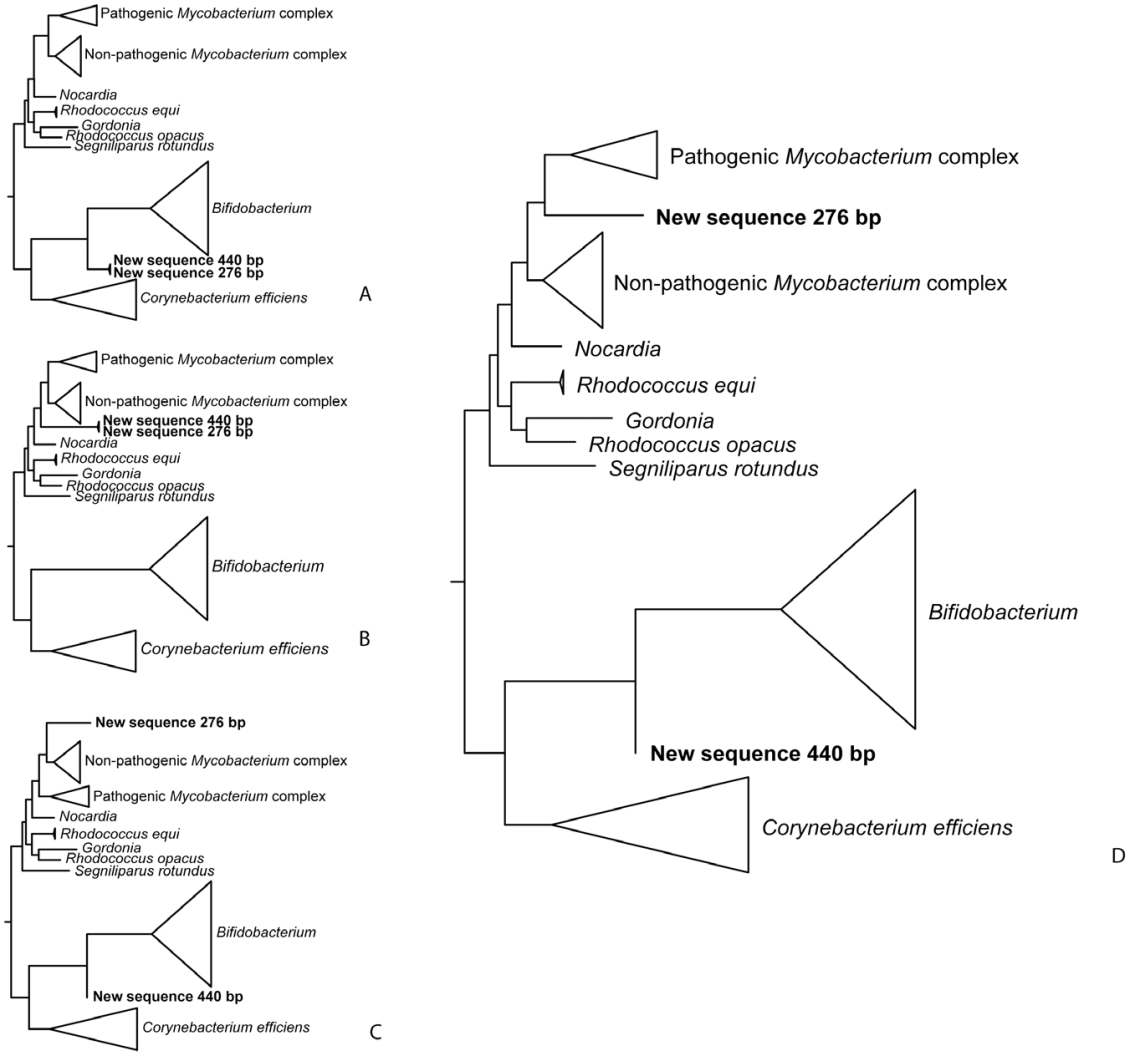
Alternative phylogenies compared to the best phylogeny. Alternative phylogenies compared to the best phylogeny obtained through maximum likelihood analyses of DNA sequence data from the Maiden’s swab samples (fig. 4) using Bayesian posterior probabilities, approximately unbiased, and weighted Shimodaira-Hasegawa tests. See table 2 for description of each alternative and test results.

To investigate the probability of misidentifying the sequences, we compared the likelihood of alternative phylogenies by examining Bayesian posterior probabilities (BPP) and using the approximately unbiased and the weighted Shimodaira-Hasegawa tests [40]. The BPP and significance of tests of alternative phylogenies were calculated by resampling site log-likelihoods in consel v1.19 [41]. Site log-likelihoods for alternative phylogenies were obtained using the *baseml* algorithm in paml v4.3 [42]. The phylogenies compared are summarized in fig. 5, and comprise alternative placements of the sequences obtained to rule out cross-contamination (sequences group with each other), or to locate the sequences more precisely in the phylogeny. The complete results of log-likelihood comparisons are shown in table 2.

**Table 2:** Maximum likelihood, Bayesian posterior probability (BPP), and significance of alternative phylogenies (for numbering, see fig. 5) using the approximately unbiased (AU) and weighted Shimodaira-Hasegawa (WSH) tests.

Phylogeny	Description	Maximum likelihood	BPP	AU	WSH
Fig. 4	Best phylogeny	−33513.8	0.762	0.652	0.982
Fig. 5A	Both sequences at base of *Bifidobacterium*	−33568.1	2E-24	0.001	0.002
Fig. 5B	Both sequences at base of *Mycobacterium*	−33560.2	6E-21	3E-04	0.001
Fig. 5C	*Mycobacterium sp. s*equence part of *smegmatis* complex	−33518.3	0.008	0.117	0.574
Fig. 5D	*Mycobacterium sp. s*equence part of *avium-bovis-tuberculosis* complex	−33515.0	0.230	0.475	0.907
Random		−47198.4	0.000	3E-78	0.000

## Results

Computed tomography (CT) scanning and radiological examinations of the Maiden revealed that all her organs, including the eyes and the brain, were intact [17]. Both radiological and visual examination revealed pathologies consistent with a range of infectious diseases: 1) a radiolucent area in the upper lobe of the right lung, 2) a mucosal enlargement of the left maxillary sinus consistent with sinusitis, 3) a zoster-like lesion on the right calf, and 4) streaks of mucus under both nostrils [20]. Similar exams on the boy revealed no lesions, and no mucosal enlargement or other signs of upper respiratory infection. To identify the proteins on the lips of both mummies, and assess the presence of pathogens we collected mouth swabs. Proteomics analyses of the mouth swabs based on high-resolution mass spectrometry revealed the presence of proteins expected in nasal secretions: serum proteins (i.e. albumin, hemoglobin and serotransferrin) in both mummies. The nasal mucus protein (PLUNC) level was three times higher in the Maiden’s sample than in the boy’s.

In addition to serum proteins, we found several proteins that are not normally present in blood or saliva, but are consistent with host immune response to infectious disease in the Maiden’s sample (see table 3). Cathepsin G is a specialized neutrophilic polymorphonuclear leukocyte serine protease found in the azurophil granules and its function has been linked to pathogenesis of diseases associated with inflammation and neutrophil infiltration of the airways, such as bacterial COPD (Chronic Obstructive Pulmonary Disease) [43], [44], [45], [46], [47], [48]. Cathepsin G and neutrophil elastase have also been found in neutrophil extracellular traps (NETs) that degrade virulence factors and kill bacteria [49]. A marker of chronic lung inflammatory diseases, α-1 antitrypsin, is a strong indicator of mycobacterial infection [50], [51], [52], [53]. It protects tissues against inflammatory, cytotoxic proteases, such as those from neutrophils. Neutrophil defensin 1 and 3 are part of the defensin family of cysteine-rich cationic proteins found in leukocytes and are specifically associated with macrophages involved in lung tissue inflammation response [54].

**Table 3:** Immune system proteins and their respective accession numbers in the swab sample of the Maiden identified by mass spectrometry.

Locus	Protein ID and description (SWISS-PROT)	Unique Peptides	Spectrum Count	Sequence Coverage
IPI00291410	Q8TDL5-1 C20orf114 Isoform 1 of Long palate, lungand nasal epithelium carcinoma-associated protein 1	2	4	4.30%
IPI00005721	P59665 DEFA1;DEFA1B Neutrophil defensin 1	9	22	31.90%
IPI00021827	P59666 DEFA3 Neutrophil defensin 3	9	22	31.90%
IPI00028064	P08311 CTSG Cathepsin G	7	12	29.00%
IPI00027769	P08246 ELANE Neutrophil elastase	4	6	11.60%
IPI00007047	P05109 S100A8 Protein S100-A8	9	20	51.60%
IPI00027462 (IPI00939362)	P06702 S100A9 Protein S100-A9	10	20	35.10%
IPI00553177 (IPI00790784)	P01009-1 SERPINA1 Isoform 1 of Alpha-1-antitrypsin	5	6	14.40%
IPI00021841	P02647 APOA1 Apolipoprotein A-I	4	5	19.90%
IPI00021854	P02652 APOA2 Apolipoprotein A-II	7	10	62.00%
IPI00022432 (IPI00646384; IPI00855916; IPI00940791)	P02766 TTR Transthyretin	3	4	22.40%
IPI00555812 (IPI00954102)	P02774-1 GC Isoform 1 of Vitamin D-binding protein	2	3	3.80%
IPI00218918 (IPI00549413)	P04083 ANXA1 Annexin A1	4	5	21.40%

Numbers in the last three columns indicate the number of unique peptides, the number of spectra observed and the sequence coverage for that particular protein (for a full list see supplemental table S1).

The proteomic analysis of the Maiden sample also uncovered two groups of proteins consistent with severe inflammation of the lungs. The first group of proteins included S100 A8/A9, apolipoprotein A1 and A2, and transthyretin. The second group of proteins included vitamin-D-binding protein (VDB), serine protease inhibitor (SERPIN) and transthyretin (TTR). The first proteins are commonly expressed in chronic and acute lung inflammations, and have been used as monitoring biomarkers for pulmonary related diseases [55], [56]. The second group of proteins is also involved in acute lung inflammation, specifically in mycobacterial infections [57]. The presence of the full complement of these proteins in the mouth swab of the Maiden provided strong evidence of response to a severe respiratory bacterial infection. The external visible symptoms and the gamut of immune response proteins obtained from the mouth swab supported the hypothesis of pulmonary infection caused by *Mycobacterium*.

The boy did not show signs of upper respiratory or pulmonary infections based on CT-scans and radiology analyses, despite the presence of blood in the mouth swab and cloth samples. For these reasons, we inferred that the boy did not have a respiratory infection, and the presence of blood was the result of trauma. Proteomic analysis of the boy’s mouth swab revealed that his α-1 antitrypsin levels were high, and neutrophil defensin levels were low. These results supported the inference that the boy was not suffering from a lung infection (see table S2). The comparison of the spectral counts in the inflammatory/immune category was highly significant (*Z* = −3.16, *P*-value = 0.0003), while the comparison for all other proteins was not significant (*Z* = 0.3602, *P*-value = 0.7206). Inflammatory and immune response proteins were elevated in the Maiden (median spectral count = 44.00 sd = 27.03) relative to the boy (median spectral count = 0.00 sd = 2.14). Levels of all other proteins detected in the samples were similar (median spectral count of Maiden = 7.00 sd = 273.33; for the boy = 10.50 sd = 274.16).

To determine the etiology of the disease, we amplified the heat-shock protein (*hsp65*) gene using *Mycobacteria*-specific primers [30], [58] and DNA extracted from the mouth swab taken from lips of the Maiden. The PCR assay followed by direct sequencing of PCR products confirmed the presence of *Mycobacterium sp.* in one of the mouth swab samples, as well as the presence of non-pathogenic *Bifidobacterium sp.* (fig. 2). The presence of *Bifidobacterium sp.* on the lips of the Maiden cannot be a result of fluid deposition during decomposition, since the bodies of the children of Llullaillaco did not decompose. We interpreted the detection of *Bifidobacterium sp.* as an indication of vomit shortly prior to her death, rather than as a result of postmortem contamination.

The position of the recovered sequence at the base of the *Mycobacterium* genus could be caused by the large amounts of missing data in the sequence (84%) relative to the genomic sequences (alignment was 1,759-bp long). Based on the best phylogeny (fig. 4), we compared alternative trees seeking to further refine the placement of our sequence (fig. 5). These comparisons ruled out cross-contamination of the *Mycobacterium sp*. sequence with DNA from non-pathogenic *Bifidobacterium sp.* (*P*≤0.002), as well as the sequence corresponding to the non-pathogenic *Mycobacterium smegmatis* complex (Bayesian posterior probability [BPP] = 0.008, more conservative approximately unbiased [AU] and weighted Shimodaira-Hasegawa [WSH] tests *P*≥0.117), but could not rule out the recovered sequence belonging to the pathogenic *Mycobacterium avium-bovis*-*tuberculosis* complex (BPP = 0.230, AU and WSH *P*≥0.475). The phylogenetic analyses indicate a higher probability for the hypothesis that the sequence corresponded to the pathogenic *Mycobacterium avium-bovis*-*tuberculosis* than to the non-pathogenic *Mycobacterium* clade (fig. 5D).

## Discussion

Ancient Andean people suffered from mycobacterial infections, as demonstrated by the presence of these pathogens in several Inca mummies preserved at the American Museum of Natural History [30]. However, mycobacteria such as *Mycobacterium tuberculosis* have the ability to persist for long periods of time without causing infection [59], [60]. Therefore, detecting the presence of the pathogen does not always indicate an active infection. In this study, we provide direct evidence of active anti-bacterial immune response at the time of death in a 500-year-old mummy. This response was significantly different from that of a putatively healthy individual preserved for the same period of time and under similar conditions. Initial radiological examination of the Maiden’s lungs showed pathological features such as over-inflation and trapped air in some areas [20], which are commonly documented in CT scans of patients affected by mycobacterial infections, and specifically the *Mycobacterium avium* and *tuberculosis* complexes [61].

The use of shotgun proteomics to detect protein remnants from ancient body fluids has many potential applications in historical and criminal sciences. We focused on samples from swabs from an archaeological specimen, but potential forensic applications include characterizing the physiological state of the source of blood in criminal cases. This technique offers a way of ascertaining whether or not an individual was sick as a result of an infection by a specific pathogen, rather than just carrying it in a latent form. Forensic proteomics offers a sensitive but less contamination-prone alternative to PCR amplification when dealing with ancient or partially degraded biological samples [4], [11], [62], [63]. Until now, immunoassays had been the only way to detect active immune response and infer infection in historical samples, but these were plagued by low specificity and sensitivity. Shotgun proteomics can play a critical role in pathological determination of the cause of disease or death in archeological, medical, and criminal cases.

## Supporting Information

Table S1 Complete list of proteins in the Maiden lip swab identified by mass spectrometry. Listed are the proteins with their respective accession number (the number in parenthesis indicates that the peptides found in the proteins are also located in other proteins). Numbers in the last two columns indicate the number of unique peptides, the number of spectra observed and the sequence coverage for that particular protein.
(DOCX)

Table S2 Comparative list of proteins list for the cloth and swab samples of the boy and the swab sample of the Maiden. Proteins are listed with accession number and description. Numbers in parentheses indicates that the peptides found in the proteins are also located in additional proteins. Numbers in the last two columns indicate the number of spectra observed in each sample. Proteins in **bold** are associated with respiratory inflammation/immune response as described in the text.
(DOCX)

## References

[1] Hawass Z, Gad YZ, Ismail S, Khairat R, Fathalla D (2010). Ancestry and Pathology in King Tutankhamun’s Family.. Journal of the American Medical Association.

[2] Raoult D, Aboudharam G, Crubezy E, Larrouy G, Ludes B (2000). Molecular identification by “suicide PCR” of *Yersinia pestis* as the agent of Medieval Black Death.. Proceedings of the National Academy of Sciences.

[3] Bos KI, Schuenemann VJ, Golding GB, Burbano HA, Waglechner N (2011). A draft genome of *Yersinia pestis* from victims of the Black Death.. Nature.

[4] Gilbert MTP, Bandelt H-J, Hofreiter M, Barnes I (2005). Assessing ancient DNA studies.. Trends in Ecology & Evolution.

[5] Cooper A, Poinar HN (2000). Ancient DNA: do it right or not at all.. Science.

[6] Timmann C, Meyer CG (2010). King Tutankhamun’s Family and Demise.. Journal of the American Medical Association 303: 2473–.

[7] Marlowe EM, Wolk DM (2006). Pathogen Detection in the Genomic Era.. Advanced Techniques in Diagnostic Microbiology.

[8] Relman DA (1999). The Search for Unrecognized Pathogens.. Science.

[9] Ye Y, Mar E-C, Tong S, Sammons S, Fang S (2010). Application of proteomics methods for pathogen discovery.. Journal of Virological Methods.

[10] Drancourt M, Raoult D (2005). Palaeomicrobiology: current issues and perspectives.. Nature Reviews Microbiology.

[11] Kricka LJ (2000). Interferences in Immunoassay–Still a Threat.. Clinical Chemistry.

[12] Washburn MP (2011). Driving biochemical discovery with quantitative proteomics.. Trends in Biochemical Sciences.

[13] Asara JM, Schweitzer MH, Freimark LM, Phillips M, Cantley LC (2007). Protein Sequences from *Mastodon* and *Tyrannosaurus rex* Revealed by Mass Spectrometry.. Science.

[14] Schweitzer MH, Zheng W, Organ CL, Avci R, Suo Z (2009). Biomolecular Characterization and Protein Sequences of the Campanian Hadrosaur *B. canadensis*.. Science.

[15] Lubec G, Afjehi-Sadat L (2007). Limitations and Pitfalls in Protein Identification by Mass Spectrometry.. Chemical Reviews.

[16] Spivey A (2009). Amplify, amplify: shotgun proteomics boosts the signal for biomarker discovery.. Environmental Health Perspectives.

[17] Ceruti MC (2003). Llullaillaco: Sacrificios y Ofrendas en un Santuario Inca de Alta Montaña.. Salta: EUCASA.

[18] Reinhard J (2005). The Ice Maiden: Inca Mummies, Mountain Gods, and Sacred Sites in the Andes.. Washington, D.C.: National Geographic Society.

[19] Wilson AS, Taylor T, Ceruti MC, Chavez JA, Reinhard J (2007). Stable isotope and DNA evidence for ritual sequences in Inca child sacrifice.. Proceedings of the National Academy of Sciences.

[20] Previgliano CH, Ceruti C, Reinhard J, Araoz FA, Diez JG (2003). Radiologic Evaluation of the Llullaillaco Mummies.. American Journal of Roentgenology.

[21] Besson M-T, Soustelle L, Birman S (2000). Selective high-affinity transport of aspartate by a *Drosophila* homologue of the excitatory amino-acid transporters.. Current Biology.

[22] Elias JE, Gygi SP (2007). Target-decoy search strategy for increased confidence in large-scale protein identifications by mass spectrometry.. Nature Methods.

[23] Eng JK, McCormack AL, Yates JR (1994). An approach to correlate tandem mass spectral data of peptides with amino acid sequences in a protein database.. Journal of the American Society for Mass Spectrometry.

[24] Tabb DL, McDonald WH, Yates JR (2002). DTA Select and Contrast: Tools for Assembling and Comparing Protein Identifications from Shotgun Proteomics.. Journal of Proteome Research.

[25] Sokal RR, Rohlf FJ (1995). Biometry: The principles and practice of statistics in biological research.. New York: Freeman.

[26] Zhang B, VerBerkmoes NC, Langston MA, Uberbacher E, Hettich RL (2006). Detecting Differential and Correlated Protein Expression in Label-Free Shotgun Proteomics.. Journal of Proteome Research.

[27] Mann HB, Whitney DR (1947). On a test of whether one of two random variables is stochastically larger than the other.. Annals of Mathematical Statistics.

[28] Hothorn T, Hornik K, Wiel MAvd, Zeileis A (2011). coin: Conditional Inference Procedures in a Permutation Test Framework..

[29] R Development Core Team (2010). R: A language and environment for statistical computing.. Vienna: R Foundation for Statistical Computing.

[30] Konomi N, Lebwohl E, Mowbray K, Tattersall I, Zhang D (2002). Detection of mycobacterial DNA in Andean mummies.. Journal of Clinical Microbiology.

[31] Park H, Jang H, Kim C, Chung B, Chang CL (2000). Detection and Identification of Mycobacteria by Amplification of the Internal Transcribed Spacer Regions with Genus- and Species-Specific PCR Primers.. Journal of Clinical Microbiology.

[32] Altschul SF, Gish W, Miller W, Myers EW, Lipman DJ (1990). Basic local alignment search tool.. Journal of Molecular Biology.

[33] Katoh K, Kuma K-i, Toh H, Miyata T (2005). MAFFT version 5: improvement in accuracy of multiple sequence alignment.. Nucleic Acids Research.

[34] Katoh K, Toh H (2008). Recent developments in the MAFFT multiple sequence alignment program.. Briefings in Bioinformatics.

[35] Stamatakis A (2006). RAxML-VI-HPC: maximum likelihood-based phylogenetic analyses with thousands of taxa and mixed models.. Bioinformatics.

[36] Stamatakis A, Ludwig T, Meier H (2005). RAxML-III: a fast program for maximum likelihood-based inference of large phylogenetic trees.. Bioinformatics.

[37] Tavaré S (1986). Some probabilistic and statistical problems on the analysis of DNA sequences.. Lectures on Mathematics in the Life Sciences.

[38] Yang Z (1994). Maximum likelihood phylogenetic estimation from DNA sequences with variable rates over sites: approximate methods.. Journal of Molecular Evolution.

[39] Felsenstein J (1985). Confidence limits on phylogenies: an approach using the bootstrap.. Evolution.

[40] Shimodaira H (2002). An approximately unbiased test of phylogenetic tree selection.. Systematic Biology.

[41] Shimodaira H, Hasegawa M (2001). Consel: for assessing the confidence of phylogenetic tree selection.. Bioinformatics.

[42] Yang Z (2007). PAML 4: Phylogenetic Analysis by Maximum Likelihood.. Molecular Biology and Evolution.

[43] Bangalore N, Travis J, Onunka VC, Pohl J, Shafer WM (1990). Identification of the primary antimicrobial domains in human neutrophil cathepsin G. Journal of Biological Chemistry.

[44] Hiemstra PS, van Wetering S, Stolk J (1998). Neutrophil serine proteinases and defensins in chronic obstructive pulmonary disease: effects on pulmonary epithelium.. European Respiratory Journal.

[45] Korkmaz B, Moreau T, Gauthier F (2008). Neutrophil elastase, proteinase 3 and cathepsin G: Physicochemical properties, activity and physiopathological functions.. Biochimie.

[46] Sommerhoff CP, Nadel JA, Basbaum CB, Caughey GH (1990). Neutrophil elastase and cathepsin G stimulate secretion from cultured bovine airway gland serous cells.. Journal of Clinical Investigation.

[47] Travis J (1988). Structure, function, and control of neutrophil proteinases.. American Journal of Medicine.

[48] Van Wetering S, Mannesse-Lazeroms SP, Dijkman JH, Hiemstra PS (1997). Effect of neutrophil serine proteinases and defensins on lung epithelial cells: modulation of cytotoxicity and IL-8 production.. Journal of Leukocyte Biology.

[49] Brinkmann V, Reichard U, Goosmann C, Fauler B, Uhlemann Y (2004). Neutrophil Extracellular Traps Kill Bacteria.. Science.

[50] Fertakis A, Archimandritis A, Tsourapas A, Douratsos A, Angelopoulos B (1977). Serum levels and alpha1-antitrypsin phenotypes in active pulmonary tuberculosis.. Acta Geneticae Medicae et Gemellologiae.

[51] Masala C, Amendolea MA, Bonini S (1976). Mucus antibodies in pulmonary tuberculosis and chronic obstructive lung disease.. The Lancet.

[52] Poh SC, Seet AM (1975). Alpha1 antitrypsin levels in chronic obstructive lung disease and pulmonary tuberculosis in Singapore.. Singapore Medical Journal.

[53] Ugajin M, Miwa S, Shirai M, Ohba H, Eifuku T (2011). Serum alpha-1-antitrypsin levels in pulmonary tuberculosis.. The European Respiratory Journal.

[54] Ganz T (2003). Defensins: antimicrobial peptides of innate immunity.. Nature Reviews Immunology.

[55] de Torre C, Ying S-X, Munson PJ, Meduri GU, Suffredini AF (2006). Proteomic analysis of inflammatory biomarkers in bronchoalveolar lavage.. Proteomics.

[56] Lau ATY, Chiu J-F (2009). Biomarkers of lung-related diseases: Current knowledge by proteomic approaches.. Journal of Cellular Physiology.

[57] Seth M, Lamont EA, Janagama HK, Widdel A, Vulchanova L (2009). Biomarker Discovery in Subclinical Mycobacterial Infections of Cattle.. PLoS ONE.

[58] Bannalikar AS, Verma R (2006). Detection of *Mycobacterium avium* & *M. tuberculosis* from human sputum cultures by PCR-RFLP analysis of hsp65 gene & pncA PCR.. Indian Journal of Medical Research.

[59] Hernandez-Pando R, Jeyanathan M, Mengistu G, Aguilar D, Orozco H (2000). Persistence of DNA from *Mycobacterium tuberculosis* in superficially normal lung tissue during latent infection.. The Lancet.

[60] Demissie A, Abebe M, Aseffa A, Rook G, Fletcher H (2004). Healthy Individuals That Control a Latent Infection with *Mycobacterium tuberculosis* Express High Levels of Th1 Cytokines and the IL-4 Antagonist IL-4Δ2.. Journal of Immunology.

[61] Wittram C, Weisbrod GL (2002). *Mycobacterium avium* complex lung disease in immunocompetent patients: radiography-CT correlation.. British Journal of Radiology.

[62] Ismail AAA, Walker PL, Cawood ML, Barth JH (2002). Interference in immunoassay is an underestimated problem.. Annals of Clinical Biochemistry.

[63] Binladen J, Wiuf C, Gilbert MTP, Bunce M, Barnett R (2006). Assessing the Fidelity of Ancient DNA Sequences Amplified From Nuclear Genes.. Genetics.

