# Supplementary material for: Detecting the Immune System Response of a 500 Year-Old Inca Mummy
Source: PLoS One. 2012 Jul 25;7(7):e41244. doi: 10.1371/journal.pone.0041244 (PMC3405130; doi:10.1371/journal.pone.0041244)
Supplement: Table S1 — Complete list of proteins in the Maiden lip swab identified by mass spectrometry. Listed are the proteins with their respective accession number (the number in parenthesis indicates that the peptides found in the proteins are also located in other proteins). Numbers in the last two columns indicate the number of unique peptides, the number of spectra observed and the sequence coverage for that particular protein. (DOCX) [file pone.0041244.s001.docx]

| **Locus** | **Protein Description (Swiss-Prot)** | **Unique Peptides** | **Spectrum Count** | **Sequence Coverage** |
| --- | --- | --- | --- | --- |
| **IPI00745872** | SWISS-PROT:P02768-1 ALB Isoform 1 of Serum albumin | 264 | 618 | 89.00% |
| **IPI00654755** | SWISS-PROT:P68871 HBB Hemoglobin subunit beta | 82 | 253 | 83.00% |
| **IPI00410714** | SWISS-PROT:P69905 HBA2;HBA1 Hemoglobin subunit alpha | 84 | 241 | 95.10% |
| **IPI00829896** | TREMBL:Q670S4 HBD Hemoglobin Lepore-Baltimore (Fragment) | 36 | 108 | 99.00% |
| **IPI00473011** | SWISS-PROT:P02042 HBD Hemoglobin subunit delta | 29 | 88 | 83.00% |
| **IPI00930351** | TREMBL:Q14477 HBD Hbbm fused globin protein (Fragment) | 20 | 63 | 75.20% |
| **IPI00022463** | SWISS-PROT:P02787 TF Serotransferrin | 35 | 58 | 36.10% |
| **IPI00384938 (IPI00876888; IPI00816314; IPI00785084; IPI00784842; IPI00784817; IPI00645363; IPI00448925; IPI00423466; IPI00423463)** | TREMBL:Q7Z351 IGHV4-31;LOC100290320;IGHG1;LOC100294459 Putative uncharacterized protein DKFZp686N02209 | 19 | 56 | 35.90% |
| **IPI00009865** | SWISS-PROT:P13645 KRT10 Keratin, type I cytoskeletal 10 | 26 | 50 | 47.60% |
| **IPI00940069** | SWISS-PROT:P01834 IGKC Ig kappa chain C region | 13 | 38 | 51.70% |
| **IPI00386524 (IPI00647704; IPI00423462)** | TREMBL:Q96DK0 IGHA1 CDNA FLJ25298 fis, clone STM07683, highly similar to Protein Tro alpha1 H,myeloma | 18 | 37 | 34.10% |
| **IPI00784865** | TREMBL:Q6P5S8 IGK@ IGK@ protein | 13 | 37 | 47.50% |
| **IPI00021439 (IPI00894498; IPI00021440)** | SWISS-PROT:P60709 ACTB Actin, cytoplasmic 1 | 15 | 32 | 38.10% |
| **IPI00021304** | SWISS-PROT:P35908 KRT2 Keratin, type II cytoskeletal 2 epidermal | 16 | 30 | 34.60% |
| **IPI00220327** | SWISS-PROT:P04264 KRT1 Keratin, type II cytoskeletal 1 | 16 | 29 | 25.20% |
| **IPI00009866** | SWISS-PROT:P13646-1 KRT13 Isoform 1 of Keratin, type I cytoskeletal 13 | 17 | 28 | 36.50% |
| **IPI00299145** | SWISS-PROT:P48668 KRT6C Keratin, type II cytoskeletal 6C | 17 | 28 | 36.70% |
| **IPI00168728 (IPI00827754; IPI00418153)** | TREMBL:Q8NF17 IGHG3 FLJ00385 protein (Fragment) | 12 | 26 | 19.30% |
| **IPI00300725** | SWISS-PROT:P02538 KRT6A Keratin, type II cytoskeletal 6A | 16 | 26 | 34.60% |
| **IPI00293665** | SWISS-PROT:P04259 KRT6B Keratin, type II cytoskeletal 6B | 15 | 25 | 31.60% |
| **IPI00887169** | TREMBL:A2MYD0 IGL@;IGLV1-44;IGLV2-11;LOC100290481;IGLV1-40;IGLC3;IGLC1;LOC100293440;IGLV3-21;IGLC2;IGLV2-14;LOC100293277;LOC100290557 Putative uncharacterized protein | 10 | 23 | 27.50% |
| **IPI00005721** | SWISS-PROT:P59665 DEFA1;DEFA1B Neutrophil defensin 1 | 9 | 22 | 31.90% |
| **IPI00021827** | SWISS-PROT:P59666 DEFA3 Neutrophil defensin 3 | 9 | 22 | 31.90% |
| **IPI00020101 (IPI00930174; IPI00794461; IPI00554798; IPI00329665; IPI00303133; IPI00152906)** | SWISS-PROT:P62807 HIST1H2BF;HIST1H2BC;HIST1H2BI;HIST1H2BG;HIST1H2BE histone cluster 1, H2bg | 12 | 21 | 73.00% |
| **IPI00007047** | SWISS-PROT:P05109 S100A8 Protein S100-A8 | 9 | 20 | 51.60% |
| **IPI00027462 (IPI00939362)** | SWISS-PROT:P06702 S100A9 Protein S100-A9 | 10 | 20 | 35.10% |
| **IPI00453473** | SWISS-PROT:P62805 HIST1H4K;HIST1H4D;HIST4H4;HIST1H4F;HIST1H4E;HIST1H4J;HIST2H4A;HIST1H4B;HIST1H4I;HIST1H4A;HIST1H4L;HIST1H4H;HIST1H4C;HIST2H4B Histone H4 | 9 | 19 | 56.30% |
| **IPI00829640** | TREMBL:Q6GMW4 IGL@;IGLV1-44;IGLV2-11;LOC100290481;IGLV1-40;IGLC3;IGLC1;LOC100293440;IGLV3-21;IGLC2;IGLV2-14;LOC100293277;LOC100290557 IGL@ protein | 7 | 19 | 30.00% |
| **IPI00290078** | SWISS-PROT:P19013 KRT4 keratin 4 | 11 | 18 | 26.80% |
| **IPI00384444** | SWISS-PROT:P02533 KRT14 Keratin, type I cytoskeletal 14 | 9 | 18 | 17.20% |
| **IPI00783987** | SWISS-PROT:P01024 C3 Complement C3 (Fragment) | 10 | 18 | 6.00% |
| **IPI00009867** | SWISS-PROT:P13647 KRT5 Keratin, type II cytoskeletal 5 | 11 | 16 | 23.40% |
| **IPI00478493 (IPI00942787; IPI00641737)** | TREMBL:Q0VAC5 HP haptoglobin isoform 2 preproprotein | 8 | 16 | 28.00% |
| **IPI00930072** | TREMBL:Q68CN4 IGHG2 Putative uncharacterized protein DKFZp686E23209 | 8 | 16 | 16.40% |
| **IPI00217963** | SWISS-PROT:P08779 KRT16 Keratin, type I cytoskeletal 16 | 8 | 15 | 13.70% |
| **IPI00930614** | TREMBL:A4FUA1 KRT13 GUCA1B protein (Fragment) | 7 | 13 | 69.30% |
| **IPI00028064** | SWISS-PROT:P08311 CTSG Cathepsin G | 7 | 12 | 29.00% |
| **IPI00021854** | SWISS-PROT:P02652 APOA2 Apolipoprotein A-II | 7 | 10 | 62.00% |
| **IPI00944677** | TREMBL:Q8N5F4 IGL@;IGLV1-44;IGLV2-11;LOC100290481;IGLV1-40;IGLC3;IGLC1;LOC100293440;IGLV3-21;IGLC2;IGLV2-14;LOC100293277;LOC100290557 IGL@ protein | 6 | 10 | 24.00% |
| **IPI00290857** | SWISS-PROT:P12035 KRT3 Keratin, type II cytoskeletal 3 | 5 | 9 | 8.30% |
| **IPI00217465 (IPI00217467; IPI00217466)** | SWISS-PROT:P16403 HIST1H1C Histone H1.2 | 4 | 8 | 18.80% |
| **IPI00008359** | SWISS-PROT:Q01546 KRT76 Keratin, type II cytoskeletal 2 oral | 4 | 7 | 6.10% |
| **IPI00026272** | SWISS-PROT:P04908 HIST1H2AB;HIST1H2AE;HIST1H2AD;HIST1H2AL;HIST1H2AK;HIST1H2AG;HIST1H2AM;HIST1H2AI;HIST1H2AJ Histone H2A type 1-B/E | 4 | 7 | 29.20% |
| **IPI00290077 (IPI00873598)** | SWISS-PROT:P19012 KRT15 Keratin, type I cytoskeletal 15 | 5 | 7 | 10.30% |
| **IPI00298497** | SWISS-PROT:P02675 FGB Fibrinogen beta chain | 4 | 7 | 11.40% |
| **IPI00027769** | SWISS-PROT:P08246 ELANE Neutrophil elastase | 4 | 6 | 11.60% |
| **IPI00217468** | SWISS-PROT:P16401 HIST1H1B Histone H1.5 | 3 | 6 | 15.00% |
| **IPI00479145** | SWISS-PROT:P08727 KRT19 Keratin, type I cytoskeletal 19 | 3 | 6 | 6.20% |
| **IPI00553177 (IPI00790784)** | SWISS-PROT:P01009-1 SERPINA1 Isoform 1 of Alpha-1-antitrypsin | 5 | 6 | 14.40% |
| **IPI00021841** | SWISS-PROT:P02647 APOA1 Apolipoprotein A-I | 4 | 5 | 19.90% |
| **IPI00030205 (IPI00954551; IPI00945366; IPI00916434; IPI00827826; IPI00387118; IPI00387115; IPI00384576)** | SWISS-PROT:P18135 IGKV3-20 Ig kappa chain V-III region HAH | 2 | 5 | 20.90% |
| **IPI00174775** | SWISS-PROT:Q86Y46-1 KRT73 Isoform 1 of Keratin, type II cytoskeletal 73 | 4 | 5 | 7.40% |
| **IPI00216457 (IPI00339274)** | SWISS-PROT:Q6FI13 HIST2H2AA4;HIST2H2AA3 Histone H2A type 2-A | 3 | 5 | 29.20% |
| **IPI00218918 (IPI00549413)** | SWISS-PROT:P04083 ANXA1 Annexin A1 | 4 | 5 | 21.40% |
| **IPI00022432 (IPI00940791; IPI00855916; IPI00646384)** | SWISS-PROT:P02766 TTR Transthyretin | 3 | 4 | 22.40% |
| **IPI00027350 (IPI00909207)** | SWISS-PROT:P32119 PRDX2 Peroxiredoxin-2 | 3 | 4 | 25.30% |
| **IPI00171611 (IPI00465070; IPI00216402)** | SWISS-PROT:Q71DI3 HIST2H3D;HIST2H3A;HIST2H3C Histone H3.2 | 3 | 4 | 19.90% |
| **IPI00219018 (IPI00797221; IPI00795257; IPI00789134; IPI00788737)** | SWISS-PROT:P04406 GAPDH Glyceraldehyde-3-phosphate dehydrogenase | 3 | 4 | 12.50% |
| **IPI00291410** | SWISS-PROT:Q8TDL5-1 C20orf114 Isoform 1 of Long palate, lung and nasal epithelium carcinoma-associated protein 1 | 2 | 4 | 4.30% |
| **IPI00022488** | SWISS-PROT:P02790 HPX Hemopexin | 2 | 3 | 3.90% |
| **IPI00215894 (IPI00924859)** | SWISS-PROT:P01042-2 KNG1 Isoform LMW of Kininogen-1 | 2 | 3 | 5.90% |
| **IPI00215983 (IPI00796435; IPI00788926)** | SWISS-PROT:P00915 CA1 Carbonic anhydrase 1 | 2 | 3 | 7.30% |
| **IPI00218192 (IPI00944960; IPI00922043; IPI00896419; IPI00896413)** | SWISS-PROT:Q14624-2 ITIH4 Isoform 2 of Inter-alpha-trypsin inhibitor heavy chain H4 | 2 | 3 | 3.10% |
| **IPI00219757 (IPI00793319; IPI00554769)** | SWISS-PROT:P09211 GSTP1 Glutathione S-transferase P | 2 | 3 | 6.20% |
| **IPI00555812 (IPI00954102)** | SWISS-PROT:P02774-1 GC Isoform 1 of Vitamin D-binding protein | 2 | 3 | 3.80% |
