# Supplementary material for: Detecting the Immune System Response of a 500 Year-Old Inca Mummy
Source: PLoS One. 2012 Jul 25;7(7):e41244. doi: 10.1371/journal.pone.0041244 (PMC3405130; doi:10.1371/journal.pone.0041244)
Supplement: Table S2 — Comparative list of proteins list for the cloth and swab samples of the boy and the swab sample of the Maiden. Proteins are listed with accession number and description. Numbers in parentheses indicates that the peptides found in the proteins are also located in additional proteins. Numbers in the last two columns indicate the number of spectra observed in each sample. Proteins in bold are associated with respiratory inflammation/immune response as described in the text. (DOCX) [file pone.0041244.s002.docx]

| **Accession Number** | **MS/MS View:Identified Proteins (115)** | **Boy (Cloth)** | **Boy**  **(Swab)** | **Maiden**  **(Swab)** |
| --- | --- | --- | --- | --- |
| IPI00220327 | SWISS-PROT:P04264 KRT1 Keratin, type II cytoskeletal 1 | 0 | 23 | 94 |
| IPI00021304 | SWISS-PROT:P35908 KRT2 Keratin, type II cytoskeletal 2 epidermal | 0 | 28 | 80 |
| IPI00382474 | SWISS-PROT:P01762 - Ig heavy chain V-III region TRO | 0 | 0 | 6 |
| IPI00171196 | ENSEMBL:ENSP00000336604 KRT13 keratin 13 isoform b | 0 | 0 | 44 |
| IPI00382490 | SWISS-PROT:P01773 - Ig heavy chain V-III region BUR | 1 | 1 | 6 |
| IPI00219018 | SWISS-PROT:P04406 GAPDH Glyceraldehyde-3-phosphate dehydrogenase | 1 | 4 | 9 |
| IPI00019359 | SWISS-PROT:P35527 KRT9 Keratin, type I cytoskeletal 9 | 1 | 0 | 7 |
| **IPI00027350 (+1)** | **SWISS-PROT:P32119 PRDX2 Peroxiredoxin-2** | **1** |  | **10** |
| IPI00179330 (+5) | SWISS-PROT:P62979 UBC;RPS27A;UBB ubiquitin and ribosomal protein S27a precursor | 2 | 1 | 3 |
| IPI00382488 | SWISS-PROT:P01771 - Ig heavy chain V-III region HIL | 2 |  | 4 |
| IPI00382482 | SWISS-PROT:P01768 - Ig heavy chain V-III region CAM | 4 | 0 | 3 |
| IPI00019591 | TREMBL:B4E1Z4 - cDNA FLJ55673, highly similar to Complement factor B | 4 | 0 | 0 |
| IPI00382483 | SWISS-PROT:P01769 - Ig heavy chain V-III region GA | 4 | 0 | 4 |
| **IPI00021827** | **SWISS-PROT:P59666 DEFA3 Neutrophil defensin 3** | **4** | **0** | **62** |
| IPI00387098 | SWISS-PROT:P01606 - Ig kappa chain V-I region OU | 5 | 0 | 3 |
| IPI00896419 | SWISS-PROT:Q14624-1 ITIH4 ITIH4 protein | 5 | 0 | 5 |
| IPI00549330 | TREMBL:Q9UL83 IGKV3D-15 Myosin-reactive immunoglobulin light chain variable region | 5 | 1 | 3 |
| **IPI00291410** | **SWISS-PROT:Q8TDL5-1 C20orf114 Isoform 1 of Long palate, lung and nasal epithelium carcinoma-associated protein 1** | **5** | **0** | **15** |
| IPI00007244 (+2) | SWISS-PROT:P05164-1 MPO Isoform H17 of Myeloperoxidase | 6 | 0 | 3 |
| IPI00847261 (+2) | H-INV:HIT000334163 - Proline-rich protein HaeIII subfamily 1 | 6 | 0 |  |
| IPI00387110 | SWISS-PROT:P01616 - Ig kappa chain V-II region MIL | 7 | 0 | 2 |
| IPI00178926 | SWISS-PROT:P01591 IGJ immunoglobulin J chain | 8 | 0 | 4 |
| IPI00473011 | SWISS-PROT:P02042 HBD Hemoglobin subunit delta | 8 | 0 | 6 |
| IPI00009650 | SWISS-PROT:P31025 LCN1 Lipocalin-1 | 9 | 8 |  |
| IPI00477090 (+2) | ENSEMBL:ENSP00000375000 IGHM 52 kDa protein | 9 | 0 | 48 |
| IPI00021439 (+1) | SWISS-PROT:P60709 ACTB Actin, cytoplasmic 1 | 10 | 15 | 101 |
| IPI00555812 (+1) | SWISS-PROT:P02774 GC Vitamin D-binding protein | 11 | 0 | 7 |
| IPI00032258 (+8) | SWISS-PROT:P0C0L4 C4A Complement C4-A | 12 | 0 | 1 |
| IPI00298497 | SWISS-PROT:P02675 FGB Fibrinogen beta chain | 14 | 0 | 22 |
| IPI00166729 | SWISS-PROT:P25311 AZGP1 alpha-2-glycoprotein 1, zinc | 14 | 0 |  |
| IPI00022431 (+1) | SWISS-PROT:P02765 AHSG cDNA FLJ55606, highly similar to Alpha-2-HS-glycoprotein | 17 | 0 | 16 |
| IPI00930684 | TREMBL:Q5EBM2 IGHG3 Putative uncharacterized protein | 17 | 0 | 29 |
| IPI00017601 | SWISS-PROT:P00450 CP Ceruloplasmin | 18 | 0 | 1 |
| IPI00855918 (+2) | ENSEMBL:ENSP00000343037 MUC5B mucin 5, subtype B, tracheobronchial | 21 | 0 | 16 |
| IPI00783987 | SWISS-PROT:P01024 C3 Complement C3 (Fragment) | 24 | 0 | 58 |
| IPI00736885 | SWISS-PROT:P01617 LOC440786 Ig kappa chain V-II region TEW | 25 | 1 | 10 |
| IPI00021885 (+1) | SWISS-PROT:P02671-1 FGA Isoform 1 of Fibrinogen alpha chain | 25 | 0 |  |
| IPI00021854 | SWISS-PROT:P02652 APOA2 Apolipoprotein A-II | 26 | 0 | 21 |
| IPI00300786 | SWISS-PROT:P04745 AMY1C;AMY2A;AMY1A;AMY1B Alpha-amylase 1 | 33 | 7 | 0 |
| IPI00829896 | TREMBL:Q670S4 HBD Hemoglobin Lepore-Baltimore (Fragment) | 34 | 0 | 31 |
| IPI00022895 | SWISS-PROT:P04217 A1BG Alpha-1B-glycoprotein | 39 | 0 | 2 |
| IPI00021841 | SWISS-PROT:P02647 APOA1 Apolipoprotein A-I | 46 | 0 | 8 |
| IPI00030205 (+1) | SWISS-PROT:P18135 IGKV3-20 Ig kappa chain V-III region HAH | 52 | 0 | 7 |
| IPI00154742 | SWISS-PROT:P01842 IGLC1;IGLV1-44;IGLV1-40;IGLV3-21;IGLV2-11;IGLV2-14;IGL@;IGLC2;IGLC3 IGL@ protein | 53 | 0 | 48 |
| IPI00298828 (+1) | SWISS-PROT:P02749 APOH Beta-2-glycoprotein 1 | 53 | 0 | 1 |
| IPI00298860 (+1) | SWISS-PROT:P02788 LTF cDNA FLJ78440, highly similar to Human lactoferrin | 54 | 0 | 5 |
| IPI00022432 (+1) | SWISS-PROT:P02766 TTR Transthyretin | 71 | 0 | 20 |
| **IPI00553177** | **SWISS-PROT:P01009-1 SERPINA1 Isoform 1 of Alpha-1-antitrypsin** | **74** | **1** | **23** |
| IPI00641737 (+1) | ENSEMBL:ENSP00000348170 HPR 47 kDa protein | 95 | 0 | 53 |
| IPI00022463 | SWISS-PROT:P02787 TF Serotransferrin | 101 | 3 | 141 |
| IPI00784817 | TREMBL:Q5EFE5 IGHG1;IGHG2;IGHV4-31;IGH@ Anti-RhD monoclonal T125 gamma1 heavy chain | 184 | 4 | 150 |
| IPI00423462 | TREMBL:Q6N092 IGHA1 Putative uncharacterized protein DKFZp686K18196 (Fragment) | 243 | 0 | 88 |
| IPI00784985 | TREMBL:Q6PJF2 IGK@ IGK@ protein | 296 | 0 | 52 |
| IPI00745872 | SWISS-PROT:P02768-1 ALB Isoform 1 of Serum albumin | 1048 | 25 | 1712 |
| IPI00410714 | SWISS-PROT:P69905 HBA1;HBA2 Hemoglobin subunit alpha | 1242 | 38 | 796 |
| IPI00654755 | SWISS-PROT:P68871 HBB Hemoglobin subunit beta | 1436 | 63 | 1070 |
| IPI00334627 (+2) | SWISS-PROT:A6NMY6 ANXA2P2 Putative annexin A2-like protein | 0 | 0 | 1 |
| IPI00290077 | SWISS-PROT:P19012 KRT15 Keratin, type I cytoskeletal 15 | 0 | 0 | 15 |
| IPI00300725 | SWISS-PROT:P02538 KRT6A Keratin, type II cytoskeletal 6A | 0 | 0 | 44 |
| IPI00397801 | SWISS-PROT:Q5D862 FLG2 Filaggrin-2 | 0 | 0 | 0 |
| IPI00450768 | SWISS-PROT:Q04695 KRT17 Keratin, type I cytoskeletal 17 | 0 | 4 | 2 |
| IPI00382470 (+1) | SWISS-PROT:P07900-2 HSP90AA1 heat shock 90kDa protein 1, alpha isoform 1 | 0 | 10 | 0 |
| IPI00792677 (+2) | TREMBL:B4DDU2 TUBA1B cDNA FLJ60097, highly similar to Tubulin alpha-ubiquitous chain | 0 | 11 | 0 |
| IPI00009865 | SWISS-PROT:P13645 KRT10 Keratin, type I cytoskeletal 10 | 0 | 19 | 155 |
| IPI00011654 (+1) | SWISS-PROT:P07437 TUBB Tubulin beta chain | 0 | 21 | 0 |
| IPI00374332 | SWISS-PROT:Q5T751 LCE1C Late cornified envelope protein 1C | 0 | 0 | 0 |
| **IPI00218918** | **SWISS-PROT:P04083 ANXA1 Annexin A1** | **0** | **0** | **9** |
| IPI00384444 | SWISS-PROT:P02533 KRT14 Keratin, type I cytoskeletal 14 | 0 | 0 | 11 |
| IPI00009867 | SWISS-PROT:P13647 KRT5 Keratin, type II cytoskeletal 5 | 0 | 0 | 13 |
| IPI00022434 | TREMBL:A6NBZ8 ALB Putative uncharacterized protein ALB | 0 | 0 | 13 |
| IPI00217465 (+2) | SWISS-PROT:P16403 HIST1H1C Histone H1.2 | 0 | 0 | 18 |
| IPI00453473 | SWISS-PROT:P62805 HIST1H4J;HIST1H4E;HIST1H4H;HIST1H4K;HIST2H4A;HIST1H4D;HIST1H4F;HIST2H4B;HIST1H4C;HIST1H4B;HIST1H4I;HIST1H4L;HIST1H4A;HIST4H4 Histone H4 | 0 | 0 | 43 |
| **IPI00028064** | **SWISS-PROT:P08311 CTSG Cathepsin G** | **0** | **0** | **44** |
| IPI00290078 | SWISS-PROT:P19013 KRT4 keratin 4 | 0 | 0 | 44 |
| IPI00020101 (+6) | SWISS-PROT:P62807 HIST1H2BI;HIST1H2BF;HIST1H2BE;HIST1H2BG;HIST1H2BC histone cluster 1, H2bg | 0 | 0 | 47 |
| **IPI00027462** | **SWISS-PROT:P06702 S100A9 Protein S100-A9** | **0** | **0** | **56** |
| **IPI00007047** | **SWISS-PROT:P05109 S100A8 Protein S100-A8** | **0** | **0** | **74** |
